# Paralysie faciale périphérique post-vaccination COVID-19: à propos d’un cas

**DOI:** 10.11604/pamj.2021.40.244.31498

**Published:** 2021-12-21

**Authors:** Smail Kharoubi

**Affiliations:** 1 Service d’Oto-Rhino-Laryngologie et Chirurgie Cervico-Faciale- Centre Hospitalier Universitaire Annaba, Faculté de Médecine, Université Badji Mokhtar, Annaba 23000, Algérie

**Keywords:** Paralysie faciale, périphérique, vaccination COVID-19, neuropathie, cas clinique, Facial palsy, peripheral, COVID-19 vaccination, neuropathy, case report

## Abstract

Il s´agit d´une observation clinique d´un patient sans contexte pathologique particulier et sans antécédents personnels ou familiaux qui consulte pour une asymétrie faciale droite deux jours après une vaccination (vaccin recombiné) contre la COVID-19. Un examen clinique complet, un bilan biologique et une imagerie par résonance magnétique (IRM) étaient sans anomalies plaidant vers l´opportunité du diagnostic d´une paralysie faciale périphérique post-vaccinale (COVID19). Une paralysie faciale périphérique dans un contexte de vaccination COVID-19 avec récupération complète est retenue.

## Abstract (en)

We conducted a clinical study of a patient with no particular medical history and without a personal or family history presenting with right facial asymmetry occurred two days after COVID-19 vaccination (recombinant vaccine). Full clinical examination, laboratory assessments and magnetic resonance imaging (MRI) were normal, suggesting the diagnosis of post-vaccine peripheral facial palsy (COVID-19). The diagnosis of peripheral facial palsy following COVID-19 vaccination with complete recovery was retained.

## Introduction

La première description de la maladie à coronavirus 2019 (COVID-19) remonte au 11 mars 2019 à partir de Wuhan. L´Organisation Mondiale de la Santé (OMS) a recensé 249 millions de cas avec 5,03 millions de décès (04 Novembre 2021). Le virus SARS-Cov-2 présente des mutations périodiques avec apparition de variants induisant des modifications épidémiologiques. Sur le plan clinique la COVID-19 présente un grand polymorphisme sémiologique qui reste dominé par un syndrome respiratoire (de gravité variable) mais également un syndrome digestif, neurologique, cardiaque, vasculaire, cutané. Plusieurs médications (antibiotiques, antipaludéens, antiviraux) ont été utilisées avec un faible niveau de preuve. Certaines sont en cours d´évaluation ou recommandées (antagonistes de l´interleukine 6) mais les résultats doivent être validés. Les mesures de protection individuelles et la vaccination constituent actuellement le fondement de la lutte contre cette pandémie. Au 18 février 2021 il existe 7 vaccins utilisés dans le monde et plus de 200 vaccins candidats en cours de mise au point, dont plus d´une soixantaine en phase de développement clinique (sous unité protéique, acide désoxyribonucléique (ADN), acide ribonucléique (ARN), vecteur viral inactivé, vecteur viral répliqué, particules virales - like, virus vivant atténué) (Tableau 1). On estime que 7,19 milliards de doses de vaccins ont été administrées dans le monde avec 3,1 milliards d´individus complètement vaccinés soit 39,7% de la population mondiale (04 Novembre 2021). La vaccination anti-COVID-19 est généralement sans complications en dehors de quelques réactions habituelles (fièvre, asthénie, myalgies, céphalées, frissons, diarrhées). D´autres complications exceptionnelles ont été rapportées: anaphylaxie, complications neurologiques (paralysies faciales, neuropathies démyélinisantes). Les centres de pharmacovigilances ont un rôle essentiel dans l´accompagnement des patients ayant bénéficié d´une vaccination afin de répertorié les accidents et incidents de toute immunisation dont certains sont tardifs et parfois limités à un nombre restreint de patients.

**Tableau 1:** principaux vaccins anti-COVID-19 et leurs caractéristiques

Vaccins	Principe	Observations
Vaccin Pfizer-BioNTech	ARNm	-2 injections espacées de 21 à 28 jours. -efficacité de 95% contre l´infection symptomatique par le SARS-CoV-2.
Vaccin Moderna	ARNm-1273	-intervalle entre injections peut aller à 12 semaines. -efficace 92% contre la COVID-19, et la protection commence 14 jours après la première dose.
Vaccin Astra Zeneca	Vecteur viral non réplicatif (adénovirus chimpanzé).	-sujets à haut risque supérieurs à 65 ans. - 2 injections de 0,5 ml avec un intervalle de 8 à 12 semaines. - une efficacité de 63,09 % contre l´infection symptomatique par le SARS-CoV-2.
Vaccin Johnson-Johnson	Vecteur adénoviral.	- 2 injections de 0,5 ml en intra musculaire ave un intervalle de 14 jours. - efficacité de 85,4% contre les formes graves de la COVID-19 et l´hospitalisation liée à cette maladie.
Vaccin Spoutnik V	Vecteurs d´adénovirus humains.	-efficacité 97,6% -2 vecteurs différents pour les deux injections. -conservation 2 et 8°.
Vaccin Spoutnik light	Vecteurs adénovirus humain.	-efficacité 79,4%. -premier composant du vaccin spoutnikV. -une seule injection suffit.
Vaccin Sinovac	Vaccin Virus SARS-Cov2 inactivé + adjuvant	-2 injections 0,5 ml intra musculaire avec un intervalle de 2 semaines. -efficacité 51% contre l´infection symptomatique SARS-Cov2 et 100% formes graves et 100% hospitalisations.
Vaccin Sinopharm	Vaccin inactivé BIBP	-0,5 ml Intra musculaire. 2 injections 3 à 4 semaines. -efficacité de 79% contre l´infection à SARS-CoV-2 symptomatique 14 jours ou plus après la deuxième dose.

## Patient et observation

**Patient information:** M. B.M âgé de 42 ans tabagique (10 paquets/an) consulte pour une asymétrie faciale droite isolée évoluant depuis 2 jours. L´histoire de la maladie ne relève pas un syndrome infectieux, ni traumatisme crânio-facial. Il n´y a pas d´antécédents personnels ou familiaux particuliers. L´anamnèse avait noté que ce patient a été vacciné contre la COVID-19 par un vaccin à vecteur viral deux jours auparavant (injection bras gauche, première dose).

**Découverte d´examen clinique:** l´examen clinique avait montré une paralysie faciale périphérique (Charles-Bell positif) grade III de House et Brackmann. La température était à 36,8°C. L´examen otologique était normal de même que celui des autres nerfs crâniens. Le reste de l´examen d’oto-rhino-laryngologie (ORL) et général était sans anomalie. La biologie avait montré une VS à 10/18 mm, protéine C-réactive (CRP) 6mg/l, les globules blancs à 7000 éléments/mm^3^, glycémie 5,60 mmol/l, urée 3 mmol/l, créatinine 90 mmol/l. Une IRM des rochers et de la fosse cérébrale postérieure n´avait pas montré de lésions ou anomalies particulières.

**Evaluation diagnostique:** au terme de ce bilan clinique et paraclinique et après analyse de l´histoire de la maladie, se séquence et l´absence de toute hypothèse différente, le diagnostic de paralysie faciale périphérique droite post-vaccinale anti-COVID-19 est retenu.

**Protocole de traitement:** une corticothérapie (prednisone 1mg/kg/jour) avait été instituée pendant 8 jours associée à des mesures de protection oculaire.

**Suivi:** un contrôle clinique le dixième jour (J10) avait montré une récupération totale de la paralysie faciale. La seconde injection a été faite la sixième semaine sans incidents majeurs en dehors d´une légère fébricule et myalgies. Un examen à trois mois était sans particularités.

**Consentement du patient:** il y a un consentement total du patient en rapport avec le contenu de ce descriptif.

## Discussion

L´incidence de la paralysie faciale périphérique (PFP) dans la population générale varie entre 15 à 30 cas/100 000 habitants/an. Les étiologies sont multiples virales, traumatiques, néoplasiques, hormonales (gravidiques), toxiques et idiopathiques (paralysie de Bell) [1]. La paralysie de Bell ou idiopathique est la plus fréquente. Peitersen en étudiant le profil évolutif des PFP rapporte une guérison à 3 semaines dans 85% des cas et entre 3 à 5 mois dans 15% des cas [1]. Au-delà de ce délai, il faut réorganiser une exploration du nerf facial (clinique, électrophysiologique et imagerie-IRM) afin de ne pas méconnaitre une lésion jusque-là non identifiée sur le trajet du nerf facial. Les paralysies faciales post-vaccinales sont connues et documentées pour plusieurs variétés de vaccins: vaccin antigrippal (plus fréquent), antidiphtérique, herpes papilloma virus, hépatite B. Cet incident demeure rare et ne dépasse pas 0,26% de l´ensemble des cas sur une période de 10 ans dans une revue publiée par Renoud [2]. Cette complication est néanmoins assez mal perçue par les patients et est parfois synonyme de gravité. L´encadrement est généralement calqué sur celui de la paralysie faciale périphérique idiopathique de type Bell avec une issue favorable.

### COVID-19 et PFP

La PFP est une complication neurologique possible de l´infection COVID-19. D´autres neuropathies y ont été associées: anosmie (nerf olfactif), dysgueusie, thrombose veineuse cérébrale, encéphalopathie, syndrome de Guillan-Barré, syndrome de Miller-Fischer et les polynévrites diffuses des nerfs crâniens. Codeluppi a constaté une augmentation des PFP de type Bell au cours de la pandémie COVID-19 avec dans 21% de ces PFP une infection récente active [3]. La survenue d´une PFP au cours de la COVID-19 se voit habituellement les trois premiers jours de l´infection mais peut également survenir après un mois du diagnostic.

Sur le plan pathogénique le virus (SARS-Cov-2) interfère avec les récepteurs de l´enzyme de conversion de l´angiotensine 2 (ACE2) qui se répartissent le long des neurones et des cellules gliales. Dubé a montré sur un modèle animal le transport dans le système nerveux de la protéine OC43 du coronavirus humain (la souche OC43 des coronavirus humains; HCoV-OC43 est un virus infectant le tractus respiratoire ayant des capacités neuro-invasives et neurotropes) [4]. Ces deux mécanismes entrainent des lésions nerveuses directes (auto-immunes) ou indirectes par action sur le vasa nervum (ischémie) ou par une démyélinisation (inflammation). La paralysie faciale est unilatérale et concerne surtout l´adulte. Theophanous a rapporté une observation d´une paralysie faciale chez un enfant âgé de 6 ans atteint de la COVID-19 [5]. Une diplégie faciale associée à une dysgueusie et un syndrome de Guillain-Barré chez un patient âgé de 44 ans a été rapportée par Khaja [6]. Le diagnostic est facile devant les signes classiques d´atteinte des voies aériennes supérieures (anosmie, toux, fièvre, asthénie) dans un contexte de pandémie ou en présence d´un cas contact. Dans tous les cas une transcriptase inverse-réaction en chaine de polymérase (RT-PCR) confirmera le diagnostic de COVID-19. L´évolution est celle d´une paralysie faciale classique de type Bell avec guérison dans la majorité des cas.

### PFP et vaccination anti-COVID-19

Aux USA la FDA (Food and Drug Administration) avait autorisé le 11 décembre 2020 la vaccination contre la COVID-19 pour un vaccin ARN messager (ARNm) BNT 162b2 (Pfizer-BioNTech) avec deux doses en injection intra-musculaire à 21 jours d´intervalle. Des effets secondaires le plus souvent mineurs ont été rapportés (fièvre, douleurs au point d´injection, asthénie, myalgies, céphalées). Quatre PFP ont été signalées chez les sujets vaccinés (21 621 personnes vaccinées) les 3, 9, 37, et 48 jours après la vaccination [7]. Depuis plusieurs observations de PFP après vaccination (ARNm) ont été publiés. Le 9 mars 2021, le registre de pharmacovigilance de l´OMS avait noté sur 133 883 cas vaccinés (vaccin ARNm), 844 (749 Pfizer-BioNTech et 95 Moderna) événements en rapport avec une paralysie faciale (0,6%) répartis comme suit: 683 paralysies faciales, 168 parésies faciales, 25 spasmes de la face, 13 cas de désordres faciaux divers [2]. Cette population était surtout féminine (67,8%), adultes (moyenne d´âge 49 ans) avec une moyenne de survenue de la PFP de 2 jours (0 à 79 jours) après une vaccination. Sur le plan évolutif il y avait une guérison totale dans 19,8%, avec séquelles dans 0,3%,une récupération subtotale dans 13,3%, sans récupération 23,9% et 42,7% de perdu de vue [2]. La PFP est généralement unilatérale, plutôt à gauche surtout à l´âge adulte. Elle peut se voir après la première ou deuxième dose de vaccin [7,8] indépendamment du coté ou se fait l´injection. Burrows avait rapporté une observation d´une PFP chez un sujet âgé de 61ans précocement dès la 5^e^ heure après la première injection (vaccin ARNm) et qui a récidiver 2 jours après la deuxième injection [8]. Cette observation est intéressante car elle montre la possibilité de PFP très précoce (5 heures contre une moyenne de 2 à 5 jours) et surtout la récidive après chaque injection ce qui renforce le lien de causalité vaccin-PFP (mécanisme immunitaire très probable). Sur le plan physiopathologique le vaccin ARNm ne contient pas d´adjuvants pouvant engendrer une réaction auto immune. Les lésions semblent surtout liées à une fixation de l´ARNm aux lipides [8].

### Vaccins non ARNm

Les effets secondaires des vaccins anti-COVID-19 non ARNm (vecteur viral) se rapprochent sur le plan clinique, physiopathologique et évolutif des vaccins habituels (antigrippal, anti-rougeole). Le rôle de l´adjuvant est suspecté (polysorbate, polyéthylène glycol) de même qu´une possible réactivation d´une infection à herpes virus simplex (HVS) [9]. La PFP est précoce (3 jours en moyenne) souvent incomplète (grade III et IV) réversibles dans la majorité des cas. Un suivi particulier est nécessaire devant un patient vacciné anti-COVID-19 aux antécédents (une ou plusieurs) de PFP ou chez qui une PFP apparait lors de la première dose de vaccin. Dans une publication récente, Wan et coll. ont rapporté les résultats d´une analyse portant sur le risque d´une PFP post-vaccination chez deux populations: une cohorte de 451 939 ayant reçu une première dose d´un vaccin de type virus inactivé (CoronaVac-Sinovac-Biotech) et une cohorte de 537 205 avec une première dose de BNT162b2 (Pfizer-BioNTech). Au 42^e^ jour ils ont noté 28 cas de PFP dans le groupe virus recombinant (CoronaVac) soit 66,9/100 000 habitants et par an et 16 cas de PFP dans le groupe ARNm (Pfizer-BioNTech) soit 42,8/ 100 000 habitants et par an. On note donc un risque légèrement élevé pour le vaccin de type virus inactivé mais avec une évolution favorable sous corticothérapie précoce à 9 mois [9].

La démarche diagnostique demeure inchangée. En cas de PFP en présence d´une infection COVID-19, l´examen clinique doit être complet en particulier otologique et nerfs crâniens. Le score de House Brackmann permet une évaluation de la gravité et un suivi. L´imagerie (IRM) n´est pas indiquée à ce stade (sauf syndromes neurologiques associés). Pour certains auteurs l´IRM peut fournir des indications pronostiques. En effet, un hyper signal du nerf facial est associé à un retard de guérison. La non récupération au-delà de 5 à 6 mois (jusqu´à 9 mois) impose une exploration (en particulier IRM). Bien que l´évolution est souvent spontanément et rapidement favorable (surtout PFP post-vaccination). Une corticothérapie, prednisone 1 mg/kg/jour pendant 10 jours avec des mesures de protection oculaires est souvent prescrite [8]. Certains auteurs réservant la corticothérapie aux formes graves de COVID-19 surtout si la PFP est modérée (grade III, IV) [8]. Les antiviraux font partis de certains protocoles: acyclovir (5 comprimés à 400 mg), valacyclovir (3 comprimés à 1 gr). Certains patients atteints de PFP suite à une infection SARS-Cov-2 ont reçu du lopinavir ou ritonavir concomitamment à leur COVID-19 sans preuve véritable d´un éventuel impact positif [10]. Un signalement au centre de pharmacovigilance est systématique. Le patient doit recevoir également une notification écrite (type fiche clinique) qui pourrait servir pour les gestes diagnostiques et thérapeutiques à venir.

## Conclusion

La PFP est un événement potentiel, bien que rare, associé à la COVID-19 (complication neurologique) ou à la vaccination anti COVID-19 (vaccins ARNm ou autres). Bien qu´étant un élément inquiétant pour le patient, il n´en demeure pas moins qu´il reste assez rare très souvent bénin et surtout mineur devant les avantages de la vaccination [8]. La prise en charge est simple avec une corticothérapie consensuelle. Les anti-viraux sont assez logiques comme traitement associé mais nécessitent une évaluation plus précise de leur impact. Il est important de notifier ces incidents pour les besoins des études de pharmacovigilance afin d´assurer la compréhension, la prise en charge rapide et la prévention (facteurs de risque à définir) de ces accidents.

## References

[1] Peitersen E (2002). Bell´s palsy: the spontaneous course of 2,500 peripheral facial Nerve palsies of different etiologies. Acta Otolaryngol.

[2] Renoud L, Khouri C, Revol B, Lepelley M, Perez J, Roustit M, Cracowski JL (2021). Association of Facial Paralysis with mRNA COVID-19 Vaccines: A Disproportionality Analysis Using the World Health Organization Pharmacovigilance Database. JAMA Intern Med.

[3] Codeluppi L, Venturelli F, Rossi J, Fasano A, Toschi G, Pacillo F, Cavallieri F, Giorgi Rossi P, Valzania F (2021). Facial palsy during the COVID-19 pandemic. Brain Behav.

[4] Dubé M, Le Coupanec A, Wong AHM, Rini JM, Desforges M, Talbot PJ (2018). Axonal transport enables neuron-to neuron propagation of human coronavirus OC43. J Virol.

[5] Theophanous C, Santoro J, Itani R (2021). Bell´s palsy in a pediatric patient with hyper IgM syndrome and severe acute respiratory syndrome coronavirus 2 (SARS-Cov-2). Brain Dev.

[6] Khaja M, Gomez GPR, Santana Y, Hernandez N, Haider A, Lara J, Elkin R (2020). A 44-year-old Hispanic man withloss of taste and bilateral facial weakness diagnosed with Guillain-Barre syndrome and Bell´s palsyassociated with SARS-Cov-2 infection treated with intravenous immunoglobulin. Am J Case Rep.

[7] Food and Drug Administration FDA Briefing Document, Pfizer-BioNTech COVID-19 vaccine, Vaccine and related biological products advisory Committee Meeting December 10,2020.

[8] Burrows A, Bartholomew T, Rudd J, Walker D (2021). Sequential contra lateral facial nerve palsies following COVID-19 vaccination first and second doses. BMJ Case Rep.

[9] Wan EYF, Chui CSL, Lai FTT, Chan EWY, Li X, Yan VKC, AL Gao (2021). Bell´s palsy following vaccination with mRNA (BNT162b2) and inactivated (CoronaVac) SARS-CoV-2 vaccines: a case series and nested case-control study. Lancet Infect Dis.

[10] Hasibi M, Seyed Ahadi M, Abdollahi H, Jafari M (2021). Protracted COVID-19 during Treatment of Facial Palsy. Case Rep Neurol Med.

